# Correction: The significance of anxiety symptoms in predicting psychosocial functioning across borderline personality traits

**DOI:** 10.1371/journal.pone.0268265

**Published:** 2022-05-04

**Authors:** The PLOS ONE Staff

The second author’s name is incorrect. The correct name is: Robinson de Jesús-Romero. The correct citation is: Howard J, de Jesús-Romero R, Peipert A, Riley T, Rutter LA, Lorenzo-Luaces L (2021) The significance of anxiety symptoms in predicting psychosocial functioning across borderline personality traits. PLoS ONE 16(1): e0245099. https://doi.org/10.1371/journal.pone.0245099

The publisher apologizes for the error.
