# Supplementary material for: The significance of anxiety symptoms in predicting psychosocial functioning across borderline personality traits
Source: PLoS One. 2021 Jan 27;16(1):e0245099. doi: 10.1371/journal.pone.0245099 (PMC7840050; doi:10.1371/journal.pone.0245099)
Supplement: S1 Table — Note. PROMIS = Patient Reported Outcomes Measurement Information System. Factor loading from an exploratory factor analysis with ordinary least squares estimation and three-factor varimax rotation. (PDF) [file pone.0245099.s001.pdf]

S1 **Table.** Exploratory factor analysis (EFA) of PROMIS depression, anxiety, and anger (N = 471)

Item	"Depression"	"Anxiety"	"Anger"
PROMIS - Depression1	0.76	0.30	0.33
PROMIS - Depression2	0.77	0.31	0.27
PROMIS - Depression3	0.76	0.36	0.33
PROMIS - Depression4	0.68	0.42	0.30
PROMIS - Depression5	0.77	0.31	0.30
PROMIS - Depression6	0.73	0.37	0.33
PROMIS - Depression7	0.66	0.44	0.36
PROMIS - Depression8	0.73	0.39	0.36
PROMIS - Anxiety1	0.34	0.64	0.28
PROMIS - Anxiety2	0.38	0.70	0.32
PROMIS - Anxiety3	0.32	0.71	0.34
PROMIS - Anxiety4	0.44	0.62	0.36
PROMIS - Anxiety5	0.35	0.76	0.37
PROMIS - Anxiety6	0.37	0.74	0.34
PROMIS - Anxiety7	0.41	0.69	0.39
PROMIS - Anger1	0.36	0.33	0.69
PROMIS - Anger2	0.36	0.41	0.72
PROMIS - Anger3	0.41	0.39	0.61
PROMIS - Anger4	0.36	0.36	0.74
PROMIS - Anger5	0.34	0.38	0.72

Note. PROMIS = Patient Reported Outcomes Measurement Information System. Factor loading from an exploratory factor analysis with ordinary least squares estimation and three-factor varimax rotation
