# Supplementary material for: The significance of anxiety symptoms in predicting psychosocial functioning across borderline personality traits
Source: PLoS One. 2021 Jan 27;16(1):e0245099. doi: 10.1371/journal.pone.0245099 (PMC7840050; doi:10.1371/journal.pone.0245099)
Supplement: S2 Table — (PDF) [file pone.0245099.s002.pdf]

S2 **Table.** Interactions between borderline personality disorder (BPD) traits and depression, anger, and anxiety and in predicting well-being and work/social adjustment while adding interactions of gender with depression, anger, anxiety, and BPD traits (n=471)

Well-being (WHO-5)	B	SE	t	*p* value
Intercept	53.71	1.22	44.09	0.00
BPD traits (MSI)	-1.55	0.46	-3.40	0.00
Depression (PROMIS)	-5.83	1.36	-4.28	0.00
Anger (PROMIS)	-4.81	1.33	-3.62	0.00
Anxiety (PROMIS)	-6.11	1.15	-5.30	0.00
Female (vs. male)	-5.62	2.11	-2.66	0.01
BPD * depression	1.08	0.39	2.79	0.01
BPD * anger	1.10	0.36	3.08	0.00
BPD * anxiety	0.87	0.38	2.33	0.02
BPD * female	-2.48	0.87	-2.85	0.00
Depression * female	-0.80	2.59	-0.31	0.76
Anger * female	-1.96	2.48	-0.79	0.43
Anxiety * female	1.88	2.28	0.83	0.41
Work and social adjustment (WSAS)	B	SE	t	*p* value
Intercept	9.05	0.53	16.95	0.00
BPD traits (MSI)	0.74	0.20	3.73	0.00
Depression (PROMIS)	3.98	0.60	6.68	0.00
Anger (PROMIS)	2.19	0.58	3.75	0.00
Anxiety (PROMIS)	1.70	0.51	3.36	0.00
Female (vs. male)	-3.02	0.93	-3.27	0.00
BPD * depression	-0.13	0.17	-0.76	0.45
BPD * anger	0.20	0.16	1.28	0.20
BPD * anxiety	0.34	0.16	2.06	0.04
BPD * female	-0.03	0.38	-0.07	0.94
Depression * female	-0.20	1.14	-0.18	0.86
Anger * female	0.64	1.09	0.58	0.56
Anxiety * female	-0.42	1.00	-0.42	0.68

Note. One woman, and two men, identified as transgender and were categorized according to their gender identity (i.e., female, and male respectively). WHO-5 = WHO-5 Well-Being Index, MSI-BPD = McLean Screening Instrument for Borderline Personality Disorder (self-report), Patient Reported Outcomes Measurement Information System, Emotional Distress

Table 2. Linear regression, with robust standard errors, predicting work and social adjustment (WSAS) from borderline personality disorder (BPD) traits, depression, anger, anxiety and their interactions while adding interactions of gender with depression, anger, anxiety, and BPD traits (n=471)

Work and social adjustment (WSAS)	B	SE	t	*p* value
Intercept	9.05	0.58	15.51	0.00
BPD traits (MSI)	0.74	0.28	2.62	0.01
Depression (PROMIS)	3.98	0.70	5.70	0.00
Anger (PROMIS)	2.19	0.69	3.18	0.00
Anxiety (PROMIS)	1.70	0.52	3.28	0.00
Female (vs. male)	-3.02	0.93	-3.26	0.00
BPD * depression	-0.13	0.21	-0.63	0.53
BPD * anger	0.20	0.19	1.06	0.29
BPD * anxiety	0.34	0.19	1.76	0.08
BPD * female	-0.03	0.51	-0.06	0.96
Depression * female	-0.20	1.37	-0.15	0.88
Anger * female	0.64	1.30	0.49	0.63
Anxiety * female	-0.42	1.06	-0.39	0.70

Note. WHO-5 = WHO-5 Well-Being Index, MSI-BPD = McLean Screening Instrument for Borderline Personality Disorder (self-report), Patient Reported Outcomes Measurement Information System, Emotional Distress
