# Supplementary material for: The significance of anxiety symptoms in predicting psychosocial functioning across borderline personality traits
Source: PLoS One. 2021 Jan 27;16(1):e0245099. doi: 10.1371/journal.pone.0245099 (PMC7840050; doi:10.1371/journal.pone.0245099)
Supplement: S3 Table — Note. VIF = variance inflation factor, WHO-5 = WHO-5 Well-Being Index, MSI-BPD = self-reported McLean Screening Instrument for Borderline Personality Disorder, Patient Reported Outcomes Measurement Information System, Emotional Distress. (PDF) [file pone.0245099.s003.pdf]

S3 Table. Interactions between depression, anger, and anxiety and borderline traits in predicting well-being and work/social adjustment (n=471), including variance inflation factors (VIFs)

Well-being (WHO-5)	B	SE	t	*p* value	β	VIF
Intercept	53.80	1.23	43.59	0.000	-0.01	
BPD traits (MSI)	-1.50	0.46	-3.26	0.001	-0.20	2.12
Depression (PROMIS)	-5.86	1.36	-4.32	0.000	-0.19	1.64
Anger (PROMIS)	-4.84	1.34	-3.61	0.000	-0.14	1.49
Anxiety (PROMIS)	-5.80	1.16	-5.00	0.000	-0.19	1.14
Female (vs. male)	-7.72	2.01	-3.85	0.000	-0.32	1.01
BPD * depression	1.12	0.39	2.86	0.004	0.14	1.53
BPD * anger	1.16	0.36	3.23	0.001	0.14	1.40
BPD * anxiety	0.84	0.38	2.24	0.026	0.10	1.20
**Work and social adjustment (WSAS)**	**B**	**SE**	**t**	***p* value**	β	**VIF**
Intercept	9.05	0.53	17.08	0.000	0.10	
BPD traits (MSI)	0.73	0.20	3.70	0.000	0.20	2.12
Depression (PROMIS)	4.07	0.58	6.98	0.000	0.32	1.64
Anger (PROMIS)	2.19	0.58	3.80	0.000	0.18	1.49
Anxiety (PROMIS)	1.72	0.50	3.45	0.001	0.16	1.14
Female (vs. male)	-3.06	0.86	-3.55	0.000	-0.26	1.01
BPD * depression	-0.13	0.17	-0.77	0.442	-0.03	1.53
BPD * anger	0.20	0.15	1.33	0.185	0.05	1.40
BPD * anxiety	0.33	0.16	2.01	0.045	0.08	1.20

Note. VIF = variance inflation factor, WHO-5 = WHO-5 Well-Being Index, MSI-BPD = self-reported McLean Screening Instrument for Borderline Personality Disorder, Patient Reported Outcomes Measurement Information System, Emotional Distress
